# Hepatic and extrahepatic metabolic modulation in hbv-related decompensated cirrhosis and acute-on-chronic liver failure

**DOI:** 10.1080/21505594.2024.2404953

**Published:** 2024-09-23

**Authors:** Zhi-Wei Li, Sheng Tu, Xia Yu, Yi-Jie Wang, Kai Gong, De-Xin Yang, Jun-Jie Yao, Hao-Tang Ren, Da-Xian Wu, Zhe-Hua Zhang, Xiao-Ling Su, Yu Wang, Zhao-Yi Pan, Rui-Hong Zhao, Ji-Fang Sheng, Yun-Qing Qiu, Yu Shi, Ze-Yu Sun

**Affiliations:** a Division of Hepatobiliary and Pancreatic Surgery, Department of Surgery, The First Affiliated Hospital, School of Medicine, Zhejiang University, Hangzhou, China; b Key Laboratory of Combined Multi-Organ Transplantation, Ministry of Public Health Key Laboratory of Organ Transplantation, Hangzhou, China; c State Key Laboratory for Diagnosis and Treatment of Infectious Diseases, National Clinical Research Center for Infectious Diseases, The First Affiliated Hospital, School of Medicine, Zhejiang University, Hangzhou, China; d Department of Infectious Diseases, The First Affiliated Hospital, School of Medicine, Zhejiang University, Hangzhou, China; e Department of Toxicology of School of Public Health, School of Medicine, Zhejiang University, Hangzhou, China; f Department of Infectious Diseases, The First Affiliated Hospital, Nanchang University, Nanchang, China; g Cellular Biology Platform, Jinan Microecological Biomedicine Shandong Laboratory, Jinan, Shandong, China

**Keywords:** Acute-on-chronic liver failure, decompensated cirrhosis, HBV, metabolomics, oxlipidomics

## Abstract

Acute-on-chronic liver failure (ACLF) and decompensated cirrhosis (DC) are life-threatening syndromes that can develop at the end-stage of chronic hepatitis B virus (HBV) infection. Both ACLF and DC are complicated by hepatic and extrahepatic pathogeneses. To better understand the compartment-specific metabolic modulations related to their pathogenesis, HBV-DC, HBV-ACLF patients, and controls (30 each) were analyzed by metabolomics using portal (Port), hepatic vein (Hep), and peripheral (Peri) serum. Compartment ratios of metabolites (Ratio_Hep/Port_, Ratio_Peri/Hep_, and Ratio_Port/Peri_) were calculated. The liver tissues (10 per group) were analyzed using transcriptomics and metabolomics. An additional 75 patients with ACLF, 20 with DC, and 20 with liver cirrhosis (LC) were used to confirm oxlipid dysregulation. Both multi-omics datasets suggest suppressed energy, amino acid, and pyrimidine metabolism in the ACLF/DC liver. The serum metabolomic variations were contributed primarily by disease rather than sampling compartments, as both HBV-ACLF and HBV-DC patients demonstrated abnormal profiles of amino acids and peptides, indoles, purines, steroids, and benzimidazoles. In ACLF/DC patients, impaired hepatic metabolism resulted in a highly correlated hepatic and portal vein serum metabolome and release of inflammatory lipids and heme metabolites from the liver. HBV-ACLF showed higher Ratio_Peri/Hep_ of extrahepatic inflammatory oxlipids, while HBV-DC patients showed higher Ratio_Port/Peri_ of gut microbial metabolites. An inflammatory oxlipid outburst was confirmed in the early stages of HBV-ACLF. The inflammatory effects of the selected oxlipids were confirmed in monocytes. These findings support a synergy between liver-specific mechanisms and systemic inflammation in ACLF/DC development, and that pro-inflammatory oxlipids are metabolic signatures of early HBV-ACLF.

## Introduction

The latest World Health Organization’s (WHO) 2024 Global Hepatitis Report alarms that the global mortality of viral hepatitis has reached 1.3 million deaths/yr and is still rising [1]. Viral hepatitis is currently ranked as the second most significant infectious cause of death worldwide and poses a serious threat to public health. Most of the viral hepatitis is related to infection with hepatitis B virus (HBV), and nearly 300 million people worldwide are chronically infected with HBV, particularly in developing countries [2]. Acute on chronic liver failure (ACLF) and decompensated cirrhosis (DC), both of which contribute significantly to the global death rate from liver illnesses, can result from chronic HBV infection as well as other chronic liver disorders. Compared to DC, ACLF is characterized by acute exacerbation of liver disease and multiple extrahepatic organ failures, which lead to high short-term mortality [3–7]. The clinical identity of ACLF remains heterogeneous, varying in the etiologies of underlying chronic liver diseases and types of precipitating events (PEs) [4,8–10]. Liver function is severely compromised during ACLF onset [11–13]. As the central metabolic organ, there must be profound changes in most hepatic metabolic pathways in patients with ACLF due to the substantial loss of hepatocytes [11,14–16]. In addition, proinflammatory PEs of ACLF, such as flare-up of hepatitis B, lead to an extrahepatic systemic cascade, in which many danger-associated molecular patterns (DAMPs) and inflammatory cytokines are released into the circulation [11,15,16].

Recent studies have shown drastic metabolomic alterations in patients with liver cirrhosis or ACLF [17–23]. The PREDICT study conducted in a European cohort [10,24] found incremental levels of systemic inflammation, along with metabolome shifts from compensated cirrhosis to decompensated cirrhosis and ACLF, and metabolite fingerprints related primarily to mitochondrial dysfunction were developed for ACLF prognosis [20,23]. Another study based on NACSELD ACLF cohorts related the poor ACLF outcome with gut microbial metabolites, including aromatic compounds, secondary bile acids (BAs), and benzoate [18]. These studies clearly show that the pathogenesis of ACLF is closely associated with complex metabolic modulation. Notably, none of these studies that measured metabolomic changes in peripheral blood samples resolved the specific metabolic reprogramming within the liver or extrahepatic compartments during DC-ACLF progression [25]. Given that both hepatic and extrahepatic events contribute to ACLF pathogenesis, we speculate that there are unique metabolic modulations in different compartments inside and outside the liver in patients with ACLF.

In the present study, we focused on patients with HBV infection, which contributed significantly to DC and ACLF cases in Asia and sub-Saharan Africa, and simultaneously investigated metabolomic modulation in the portal vein, hepatic vein, and peripheral circulation of DC and ACLF patients using high-coverage UPLC-HRMS metabolomics. The correlation between hepatic tissue metabolomics and transcriptomics in DC and ACLF patients also investigated. This study aims to capture DC-ACLF-related metabolic changes in the intrahepatic and extrahepatic compartments of HBV patients and to assess how hepatic and systemic metabolic modulations were linked to HBV-DC/ACLF pathogenesis.

## Materials and methods

### Study design and patients’ enrollment

This study consecutively enrolled 30 patients with HBV-related ACLF and 30 patients with HBV-related decompensated cirrhosis (DC) who were referred between 1 November 2018, and 31 May 2019, to the liver transplantation center of The First Affiliated Hospital of Zhejiang University and underwent liver transplantation. The control group included 30 patients without underlying chronic liver diseases who were referred to the hospital during the same period and underwent partial hepatectomy for hepatic hemangioma, gallbladder carcinoma, and hepatic metastasis of colorectal cancer. The demographic and clinical information is shown in Table 1. This study was approved by the ethics committee of the First Affiliated Hospital of Zhejiang University School of Medicine (reference number: 2018244) and was conducted according to the principles of the Declaration of Helsinki. Written consent was obtained from each patient or authorized representative(s).

**Table 1.** Clinical characteristics of enrolled patients.

Variables	ACLF (n = 30)	Decompensated cirrhosis (n = 30)	controls (n = 30)	P value*
Age (years)	44.4 ± 10.5	48.0 ± 10.9	54.7 ± 14.4	0.199
Sex (M/F)	28/2	23/7	20/10	0.148†
Underlying cirrhosis (%)	30 (100%)	30 (100%)	0 (0%)	–
**ACLF grades**				
ACLF-1	8(26.7%)	–	–	–
ACLF-2	12(40.0%)	–	–	–
ACLF-3	10(33.3%)	–	–	-
**Complications**				
Ascites	20(66.7%)	22(73.3%)	–	0.389†
Gastroenterological bleedings	5(16.7%)	12(40.0%)	–	0.042†
Hepatic encephalopathy	22(73.3%)	2(6.7%)	–	<0.001†
Bacterial infections	11(36.7%)	7(23.3%)	–	0.199†
HRS	4(13.3%)	0(0.0%)	–	0.056†
**Laboratory parameters**				
WBC	8.6 ± 3.6	3.7 ± 2.4	6.2 ± 2.6	<0.001
ALT (U/L)	774.6 ± 839.8	32.3 ± 30.9	26.9 ± 20.2	<0.001
AST (U/L)	496.4 ± 590.2	44.9 ± 36.1	24.3 ± 10.3	<0.001
ALB (g/L)	32.9 ± 5.0	32.3 ± 6.4	43.5 ± 5.3	<0.001
Cr (μmol/L)	68 ± 70.2	67 ± 18.2	68.6 ± 11.6	0.245
Bilirubin (μmol/L)	300.9 ± 106.5	40.9 ± 29.0	12.1 ± 6.6	<0.001
INR	3.2 ± 1.2	1.4 ± 0.2	1.0 ± 0.1	<0.001
HBV DNA (log10 IU/mL)	5.09 ± 2.28	2.49 ± 1.09	2.25 ± 0.66	<0.001
HBsAg (IU/mL)	11043.31 ± 27425.27	550.38 ± 623.88	584.28 ± 1667.8	<0.001
HBeAg positive	11 (37.9%)	4 (13.3%)	4 (14.3%)	0.061
**Severity scores**				
MELD score	31.3 ± 5.2	13.1 ± 3.4	7.0 ± 1.0	<0.001
CLIF-SOFA score	11.3 ± 3.6	3.8 ± 2.8	0.3 ± 0.7	<0.001
**Organ failure**				
Liver failure	27(90.0%)	0(0.0%)	0 (0.0%)	<0.001†
Renal failure	7(23.3%)	0(0.0%)	0 (0.0%)	<0.001†
Circulation failure	2(6.7%)	1(3.3%)	0 (0.0%)	0.146†
Respiratory failure	5(16.7%)	1(3.3%)	0 (0.0%)	0.008†
Coagulation failure	26(86.7%)	0(0.0%)	0 (0.0%)	<0.001†
Brain failure	14(46.7%)	1(3.3%)	0 (0.0%)	<0.001†

*One-way ANOVA unless otherwise indicated. † Chi-square test.

AD, acute decompensation; SDC, stable decompensated cirrhosis; UDC, unstable decompensated cirrhosis; HRS, hepatorenal syndrome; ALT, alanine aminotransferase; ALT, alanine aminotransferase; AST, aspartate aminotransferase; ALB, albumin; Cr, creatinine; HBeAg, hepatitis B e antigen. INR, international normalized ratio; MELD, Model for end-stage liver disease; CLIF-SOFA, Chronic Liver Failure Sequential Organ Failure Assessment.

The diagnosis of HBV-ACLF in the study met the Chinese Group on the Study of Severe Hepatitis B (COSSH) criteria and had a high overlap with The Asian Pacific Association for the Study of the Liver (APASL) criteria as well as the European Association for the Study of the Liver-Chronic Liver Failure (EASL-CLIF) criteria. The exclusion criteria, diagnosis of cirrhosis, and routine clinical and biomedical test details can be found in Supplementary Materials. The MELD and CLIF-SOFA scores were calculated for patients with ACLF and DC.

### Sample collection

Blood samples from the peripheral, portal, and hepatic veins were collected from each patient. Peripheral blood was collected before anesthesia. The portal and hepatic vein blood samples were obtained by needle puncture of the extrahepatic parts after exposure to hilar block (control group) or de-segmentation (ACLF and DC groups). Diseased liver tissues from patients with ACLF and DC were obtained during liver transplantation. For the control group, adjacent normal liver tissues were obtained from the resected lesions. The procedures for serum and tissue preparation are provided in the Supplementary Materials.

### Untargeted metabolomics analysis

Metabolites were extracted from the hepatic vein (Hep), portal vein (Port), and peripheral vein (Peri) serum samples, or liver tissues. Metabolome fingerprinting was performed on a Vanquish UPLC system coupled with an Orbitrap Q-Exactive HFX (Thermo Fisher) high-resolution mass spectrometer (HRMS) platform. Detailed UPLC- HRMS parameters and data processing procedures are provided in the Supporting Information. In all comparisons, Student’s *t*-test with BH adjusted FDR < 0.05, and fold-change >1.5 were used to select significantly altered metabolites.

From serum metabolomics data collected at three different locations (Hep, Port, and Peri), the pair-wise intensity ratio of each metabolite (m) was calculated by dividing the normalized UPLC-HRMS intensities (NormInt) between locations in each individual (i):

$$\mathrm{Rati}o_{\mathrm{Hep}/P\mathrm{ort},\;m,i}=\;\mathrm{NormIn}t_{\mathrm{Hep},m,i}/\mathrm{NormIn}t_{\mathrm{Port},m,i}$$

$$\mathrm{Rati}o_{\mathrm{Peri}/Hep,m,i}=\;\mathrm{NormIn}t_{\mathrm{Peri},m,i}/\mathrm{NormIn}t_{\mathrm{Hep},m,i};$$

$$\mathrm{Rati}o_{\mathrm{Port}/P\mathrm{eri},m,i}=\;\mathrm{NormIn}t_{\mathrm{Port},m,i}/\mathrm{NormIn}t_{\mathrm{Peri},m,i};$$

### RNA-seq analysis

Liver RNA-seq was performed using the Illumina NovaSeq platform. Reads were mapped to the human reference genome and the FPKM for each gene was quantified using featureCounts (v1.5.0). In this study, only protein-coding genes (PCGs) were used for analysis. Differential expression analysis was performed using edgeR. P-values <0.05, and fold-change ≥2 were set as the threshold to select differentially expressed genes (DEGs). The detailed method is provided in the Supplementary Materials.

### Targeted oxlipidomics analysis

Oxlipid profiles of peripheral serum samples were analyzed using a 6500+ Qtrap (Sciex) tandem mass spectrometer. A list of 116 lipids resulted in robust signals across all samples and was used for inter-group comparisons. Detailed information on the targeted oxlipid species is provided in Supplementary Table 5.

### Oxlipid stimulation on macrophage model

Oxlipids showing an increase in ACLF patients, including thromboxane B2 (TXB2), 12-hydroxyicosapentaenoic acid (12HEPE), and 12-oxoicosatetraenoic acid (12OxOETE, or 12KETE) at concentrations of 0.1, 1, and 10 μM were used to treat THP-1 monocytes pre-activated with PMA or LPS-PMA. Cell-secreted IL-6, IL-10, and TNFα levels were measured using a cytometric bead array. The experimental details can be found in the Supplementary Materials.

### Multivariate modeling and statistical analyses

Quantitative variables are expressed as mean ± standard deviation, and qualitative variables are expressed as absolute and relative frequencies. Continuous clinical and biochemical data were compared using one-way ANOVA or Student’s *t*-test, while categorical data were compared using the chi-square test. Statistical significance was set at *p* < 0.05. Overall variation within each set of omics data was summarized by principal component analysis (PCA). The joint variation between the hepatic metabolomics and transcriptomics data sets was extracted using two-way orthogonal partial least square analysis (O2PLS). All PCA and O2PLS models were developed using SIMCA-P v14.1 (Umetrics AB, Sweden). For genes and metabolites, as well as three ratios of metabolites between locations, inter-group comparisons (ACLF/control, DC/control, and ACLF/DC) were performed using Student’s *t*-test, and fold-changes were calculated using the ratio of means.

## Results

### Study cohort characteristics

**Figure 1.**
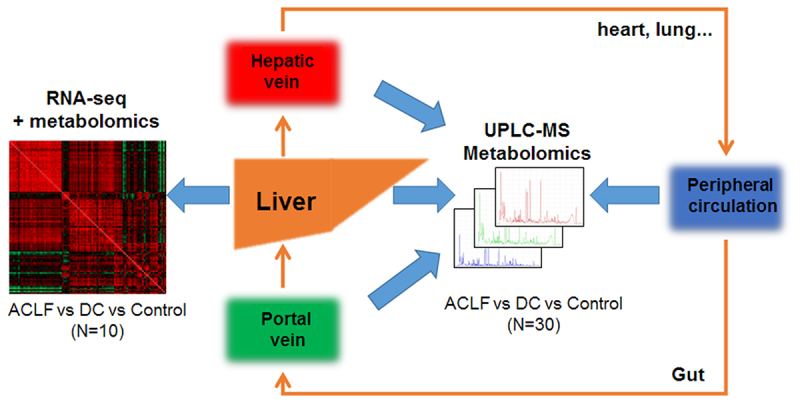
Overall schematic workflow of multi-omics approach designed to simultaneously investigate liver metabolomics and transcriptomics, as well as serum metabolomics from portal vein, hepatic vein and peripheral circulation of DC and ACLF patients.

A total of 30 hBV-ACLF patients, 30 hBV-DC patients, and 30 controls were enrolled in the serum metabolomics study, and liver tissue samples from 10 subjects in each group were used for RNA sequencing. The overall design of this multi-omics study is shown in Figure 1. Patients in all groups were matched with respect to age and sex (Table 1). More than half of the HBV-ACLF patients developed hepatic encephalopathy (HE). As expected, HBV-ACLF patients had significantly higher levels of WBC, ALT, AST, and INR than the other two groups. The Cr level was higher; therefore, more renal failure cases were found in the HBV-ACLF group. The MELD and CLIF-SOFA scores were also higher in HBV-ACLF patients than in HBV-DC patients.

### Joint alteration of hepatic transcriptome and metabolome

**Figure 2.**
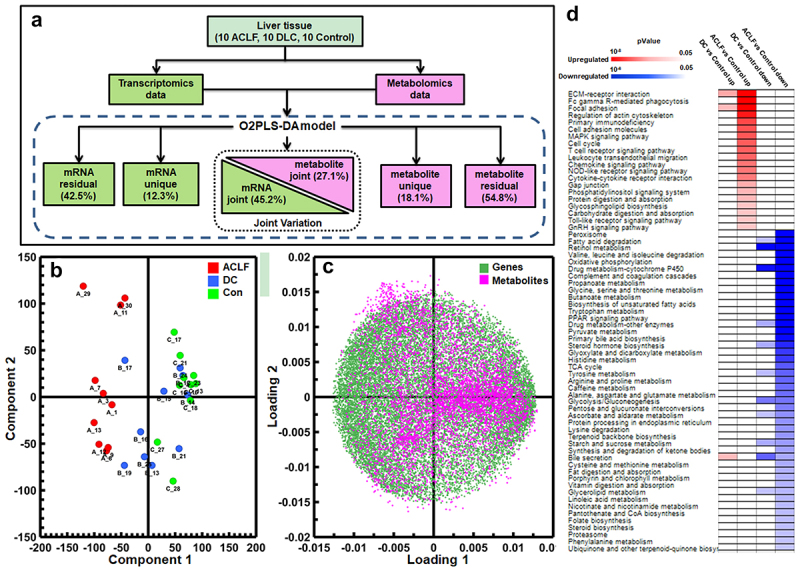
Characterization of liver metabolomics and transcriptomics in ACLF, DC and control patients. (a) schematic workflow of O2PLS modeling to unveil metabolomics and transcriptomics joint variation. (b) O2PLS score plot showing metabolomics and transcriptomics joint variation was related to disease progression, majorly modeled by component 1. (c) O2PLS loading plot showing correlation each metabolite and gene to component 1 (loading 1) and component 2 (loading 2), therefore reflect their contribution to the sample inter-group separation. (d) key pathway components significantly altered in liver tissue during ACLF/DC progression.

A total of 16,720 genes and 5879 metabolites were identified across all tissue samples by RNA sequencing and LC-MS analysis (Supplementary Tables S1 and S2). To unveil joint alterations between the two datasets, an O2PLS model (Figure 2a) was constructed, with 78% goodness- of-fit (R^2^Y) and a goodness of prediction (Q^2^) of 62%. The model revealed that 45.2% of the transcriptomic variance and 27.1% of the metabolomic variance were correlated and contributed to the differentiation of patients (Figure 2b). In addition, 12.3% unique variance in the transcriptomic data and 18.1% unique variance in the metabolomic data were related to inter-group differences. The contribution of each gene and metabolite to the inter group differences in O2PLS model were visualied in loading plot (Figure 2c).

Compared with controls, 490 and 194 DEGs were up-regulated and down-regulated in DC patients, respectively, while 4602 and 3321 DEGs were up-regulated and downregulated in HBV-ACLF patients, respectively. In addition, when compared with DC patients, 2548 up-regulated and 2423 down-regulated DEGs were found in HBV-ACLF patients (Supplementary Table S1). Pathway analysis suggested that the up-regulated genes in HBV-ACLF patients were involved in tissue microenvironment remodeling (ECM-receptor interaction, focal adhesion, gap junction, regulation of actin cytoskeleton) and immunological responses or inflammation (Toll-like receptor signaling pathway, cytokine-cytokine receptor interaction, chemokine signaling pathway, leukocyte transendothelial migration, T cell receptor signaling pathway, Fc gamma R-mediated phagocytosis), whereas downregulated genes suggested compromised central energy metabolism (mitochondrial oxidative phosphorylation, glycolysis, pentose and glucuronate conversion, TCA cycle, fatty acid beta-oxidation, butanoate/propanoate, and amino acid metabolism). Downregulated genes were also related to the metabolism of steroid hormones, bile acids, vitamins, folate, porphyrin, and xenobiotics. In contrast, HBV-DC patients showed a significantly less modulated transcriptome, with few increasing genes related to ECM-receptor interaction and focal adhesion, and decreased genes related to glycolysis and metabolism of amino acids, glycerolipid, steroids, and bile acids (Figure 2d).

Compared with controls, 565 increased and 265 decreased metabolites were found in HBV-DC livers, while 1232 increased and 1893 decreased metabolites were found in HBV-ACLF livers. There were 245 up- regulated and 192 downregulated components also showing joint modulation with the transcriptomic data (VIP >1 in the O2PLS-DA model). These include bilirubin, secondary BAs (7α-hydroxy-3-oxochol-4-en-24-oic acid, 3-oxo-4,6-choladienoic acid), and the mitochondrial toxin dibutyl phthalate, all of which showed substantially increased levels (>5 fold) in HBV-ACLF patients. Increasing level of hepatoxic hematoporphyrin, acylcarnitines (stearidonyl, tetradecanoyl, dodecanoyl, 12-hydroxy-12-octadecanoyl, 3-hydroxyhexadecadienoyl, 3-hydroxyhexadecanoyl, and trans-2-tetradecenoyl carnitine, hexadecanedioic acid mono-L-carnitine ester) and fatty acid derivatives (3-oxotetradecanoic acid, 10-nitrolinoleate, N-myristoylglycine, 2-hydroxycaproic acid, stearoylethanolamide, 3-hydroxydecanoic acid, 2-linoleoylglycerol, 2-arachidonyl glyceryl ether, adrenic acid, 1-hexadecanal, 2-hydroxypropyl stearate, 1-glyceryl stearate, desmeninol, tetracosahexaenoic acid) were also found in ACLF/DC patients. Notably, medium-chain hydroxyl acids, such as 3-hydroxydecanoic acid and 2-hydroxycaproic acid, which are both known neurotoxins [25–27], can be the culprit for encephalopathy. In parallel with the transcriptomic data, decreased levels of intermediates or metabolites in the central energy pathway (glucose, gluconolactone, gluconic acid, erythrose 4-phosphate, erythronic acid, glyceraldehyde 3-phosphate, glycerol 3-phosphate, NAD), pentose pathway (phosphoribosyl pyrophosphate, sedoheptulose 7-phosphate, ribulose 5-phosphate), amino acid metabolism (3-hydroxyanthranilic acid, S-acetyldihydrolipoamide, glutaric acid), and pyrimidine metabolism (uridine 5’-diphosphate, CMP-sialic acid, uridine, 5-methylcytidine) were observed in ACLF/DC patients.

### Overall characteristics of HBV-DC and HBV-ACLF serum metabolome

**Figure 3.**
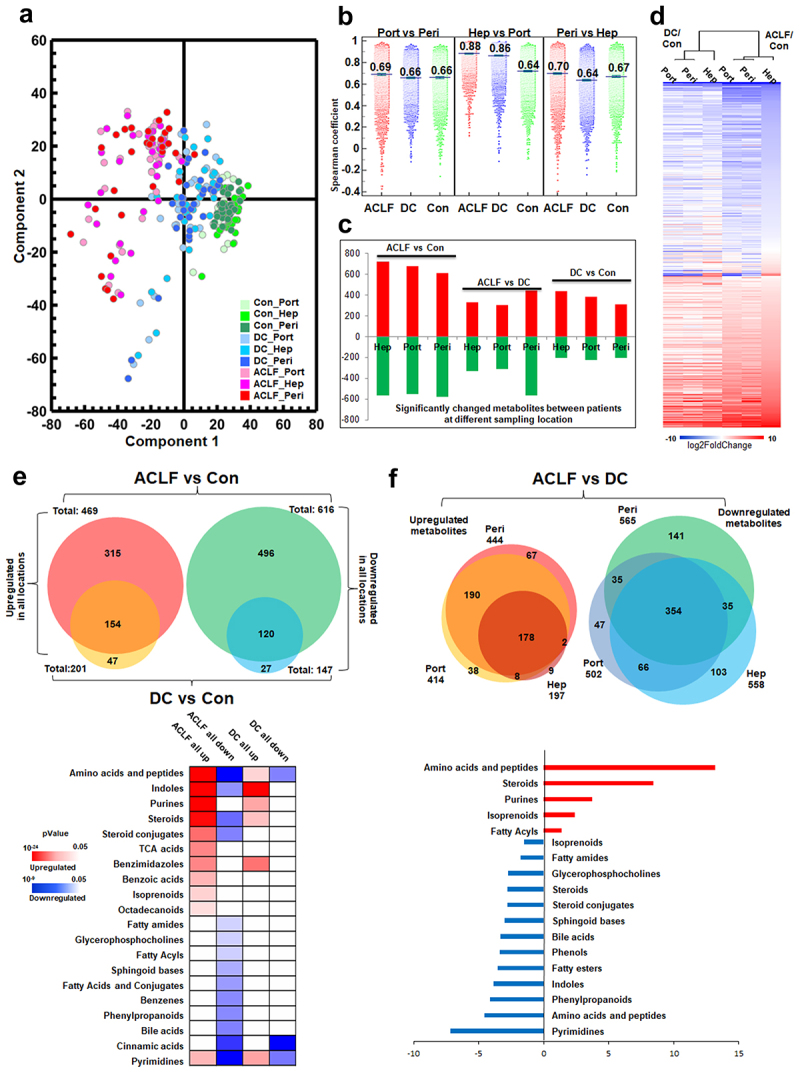
Characterization of serum metabolomics at portal vein (port), hepatic vein (hep) and peripheral circulation (Peri) from ACLF, DC and control patients. (a) overall PCA plot showing overall inter-group differences of serum metabolome were majorly attributed to disease progression. (b) comparison of spearman coefficient of all metabolites between different locations showing the similarity of portal vein and hepatic vein serum metabolite profiles were higher in DC and ACLF patients. (c) summary of up- or down-regulated metabolites in each location between patient groups. (d) heatmap of significantly altered metabolites in DC and ACLF as compared to control samples. The averaged LC-MS intensity ratio of each metabolite in DC or ACLF samples versus control samples were Log2 transformed before hierarchical clustering by Euclidean distance. (e) chemical category enrichment analysis of metabolites that shown significantly alterations comparing DC or ACLF versus controls across all 3 locations, enrichment significance (p values) were visualized by heatmap. (f) number of metabolites that shown significantly alterations comparing ACLF versus DC in all 3 locations while their chemical attributes were summarized in barplot.

The overall PCA model based on 3267 serum metabolites identified in all three locations (Supplementary Table S3) demonstrated a clear separation of patients at all sampling locations (R^2^X = 0.797, Q^2^ = 0.61, Figure 3a). Compared with the marked inter-group differences (Supplementary Figure S1), the metabolome profiles at different locations from the same individual were highly similar (Figure 3a, Supplementary Figure S2). Therefore, the overall metabolomic variations were primarily related to disease states. Pairwise covariance analyses revealed that metabolites measured between locations (Hep vs. Port, Peri vs. Hep, Port vs. Peri) were positively correlated (Corr. Coef ~ 0.6; Figure 3b in all groups. Interestingly, the overall correlation coefficient of Hep vs Port from HBV-DC or HBV-ACLF patients significantly increased to 0.86 or 0.88 (Figure 3b), indicating the absence of hepatic metabolic influence in DC/ACLF patients. In other words, blood metabolites simply “pipe-through” the ACLF/DC liver without being modified (converted/absorbed/released).

Given the similarity of metabolome across the three locations, the screening criterion of the common metabolic shift between HBV-ACLF and HBV-DC patients was set as metabolites with > 1.5 fold-change and BH-FDR <0.05 in all locations. We observed > 300 upregulated and > 200 downregulated metabolites in the HBV-DC vs. Con comparison, while there were > 600 upregulated and > 500 downregulated metabolites in the HBV-ACLF vs. Con comparison (Figure 3c). The overall metabolomic shift was similar in HBV-ACLF and HBV-DC patients, but more substantial in HBV-ACLF patients. The incremental DC-ACLF disease progression can be evidenced by the fact that the fold changes of metabolites in the HBV-ACLF/control comparison were generally more substantial than those in the HBV-DC/controls (Figure 3d). Moreover, we found that most metabolites changed in the HBV-DC/control comparison overlapped with that of the HBV-ACLF/control comparison, and no metabolites showed opposite changing directions (Figure 3e). Metabolites that were commonly upregulated in HBV-ACLF and HBV-DC patients included AAs and oligopeptides, indoles, purines, steroids, and benzimidazoles, while the most downregulated metabolites also included AAs, oligopeptides, cinnamic acids, and pyrimidines (Figure 3e).

**Figure 4.**
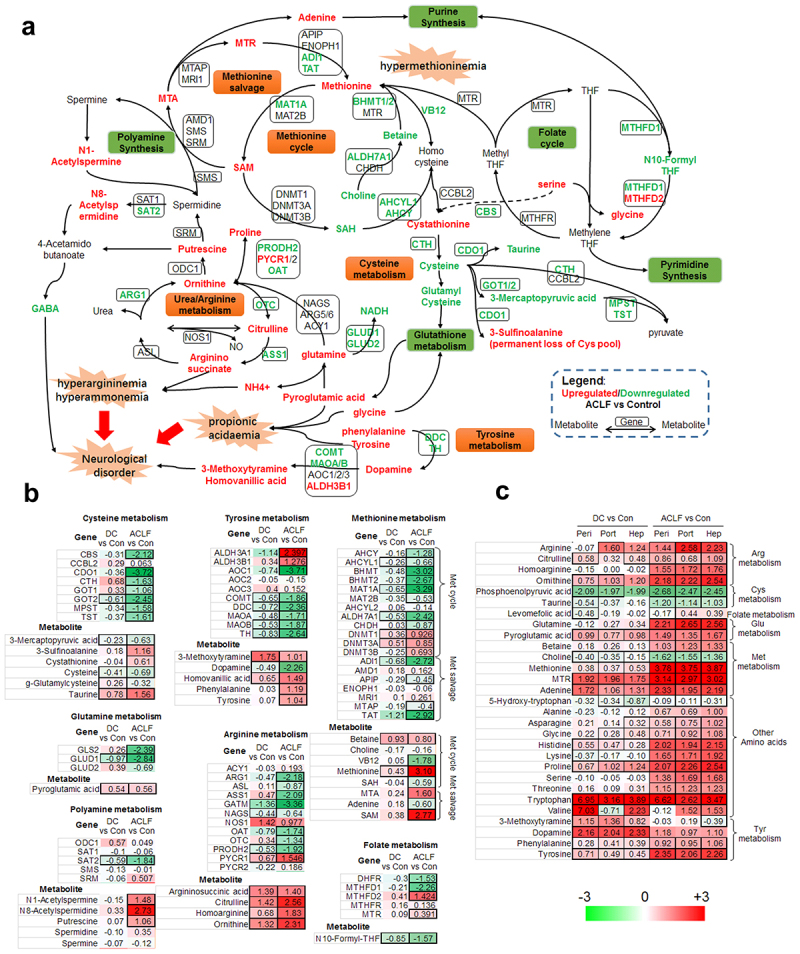
Metabolic network connected amino acids, urea, pyrimidine, purine and polyamine metabolism (a). Key enzymes in each metabolic conversion step were encircled by box. Red and green represent up- or down-regulated metabolites or genes in ACLF patient compared to controls; while black represent no significant changes. GABA, γ-aminobutyric acid; GSH, reduced glutathione; GSSG, oxidized glutathione; MTA, 5”-deoxy-5”-(methylthio)adenosine; MTR, methylthioribose; SAM, S-adenosylmethionine; SAH, S-adenosylhomocysteine; THF, tetrahydrofolate. Alteration of hepatic metabolite and genes between patients were summarized by heatmaps grouped by amino acid metabolism pathways (b), while their corresponding levels in serum samples were summarized in (c). In all heatmaps, up- or down-regulated genes or metabolites were colored by red or green shades, comparisons reached statistical significance (student’s *t*-test with BH adjust FDR < 0.05) were framed by solid border.

Although most metabolites showed similar alterations in HBV-ACLF and HBV-DC patients, there were 178 upregulated and 354 downregulated metabolites in all three locations, distinguishing HBV-ACLF from HBV-DC patients (Figure 3f). In particular, higher levels of methionine, lysine, serine, glutamine, tyrosine, and AA derivatives, such as pipecolic acid, N-acetylvanilalanine, N-acetyl-tyrosine, and homoarginine, were found across all locations in patients with HBV-ACLF (Figure 4). Moreover, the levels of steroids, TCA acids, benzoic acids, isoprenoids, and octadecanoids and lower levels of pyrimidines (uracil, deoxyuridine, cytidine), FAs, fatty amides, fatty acyls, glycerophosphocholines, sphingoid bases, benzenes, phenylpropanoids, and BAs were also higher in HBV-ACLF patients than in HBV-DC patients.

### Metabolomic shift between compartments in HBV-DC and HBV-ACLF patients

Since the metabolomic changes between compartments within each individual were disproportionally smaller than those between patients, we then focused on three pair-wise ratios measuring the subtle changes along the circulation for each metabolite to avoid interference from the huge inter-individual variance (Supplementary Table S4).

**Figure 5.**
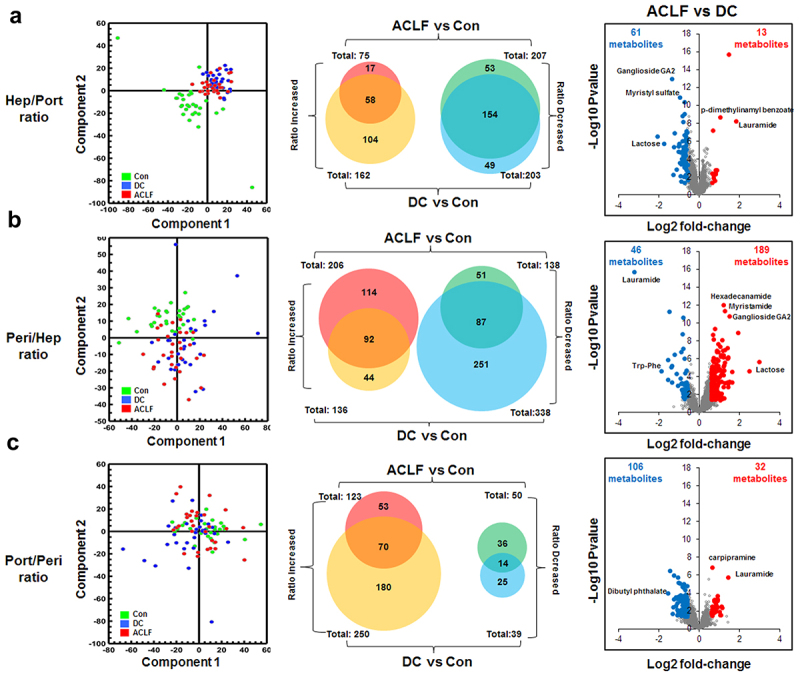
Characterization of serum metabolomics shift between locations by Ratio_Hep/Port_ (a), Ratio_Port/Peri_ (b), and Ratio_Peri/Hep_ (c). The left panel includes PCA plots showing overall shift of 3 pair-wise ratios attributed to disease progression. The middle panel summarizes up- or down-regulated metabolites in each pair-wise ratio between ACLF vs con or DC vs con. Right panel includes volcano plots showing significantly changed metabolites (student’s *t*-test with BH adjust FDR < 0.05 and fold-change >1.5) between ACLF and DC.

Ideally, the Ratio_Hep/Port_ should be directly influenced by hepatic metabolism. Compared to controls, HBV-DC and HBV-ACLF patients had 207 and 203 metabolites, respectively, showing a decreasing Ratio_Hep/Port_ ratio, while 75 and 162 with increasing Ratio_Hep/Port_ (Figure 5a). HBV-DC and HBV-ACLF livers released fewer FAs (i.e. decreasing Ratio_Hep/Port_ of myristic acid, dihydrolipoic acid, caprylic acid, 10-nitrolinoleic acid, myristoleic acid, 5-dodecenoic acid) while releasing more acylcarnitine compounds (i.e. Ratio_Hep/Port_ of hexadecanedioic acid mono-L-carnitine ester, 3-methylcrotonylglycine, tetradecanoylcarnitine), and FA amide (hexadecanamide, myristamide, lauramide, oleamide, linoleamide), indicating that hepatic beta-oxidation of FAs is impaired in ACLF/DC patients. In addition, failed hepatic heme metabolism in ACLF/DC patients is evidenced by higher Ratio_Hep/Port_ of hepatoxic hematoporphyrin and bilirubin as well as lower Ratio_Hep/Port_ of bilirubin glucuronide, which is supposed to be converted by the liver for detoxification. The ACLF/DC liver also released more BAs into circulation (higher Ratio_Hep/Port_ of CA, TCA, GCA), which are supposed to be secreted into bile instead. Enhanced hepatic release of multiple LysoPC/PE and PC(O-16:0/2:0) species, also known as platelet-activating factors (PAF), was found in ACLF/DC patients.

Alterations in the Ratio_Peri/Hep_ primarily reflected extrahepatic events. Compared to the controls, there were 206 and 138 metabolites with increasing or decreasing Ratio_Peri/Hep_ in HBV-ACLF patients, respectively. Unexpectedly, when compared to controls, HBV-DC patients also had 136 and 338 metabolites with increasing or decreasing Ratio_Peri/Hep_, suggesting there were extrahepatic events in HBV-DC patients, albeit different from those in HBV-ACLF patients (Figure 5b). Mixed Ratio_Peri/Hep_ alterations of FAs, lysophosphatidylethanolamines (lysoPEs), lysophosphatidylcholine (LysoPC), and phosphosphingolipids (PSs) can be found in both HBV-DC and HBV-ACLF patients. Focusing on metabolites that differ HBV-ACLF from HBV-DC patients, we observed higher Ratio_Peri/Hep_ of inflammatory oxlipid mediators including prostaglandin E2 (PGE2), leukotriene B4 (LTB4), 12S-hydroxy-5Z,8E,10E-heptadecatrienoic acid (12-HHTrE), 14,15-dihydroxyeicosatrienoic acid (14,15-DiHETrE), 13-hydroperoxylinoleic acid (13-HPOD), 8,9-epoxyeicosatrienoic acid (8,9-EET) and 13-keto-9,11-octadecadienoic acid (13-OxoODE), possibly as the result of systemic inflammation and oxidative stress.

Ratio_Port/Peri_ measures pre-hepatic metabolic alterations that are likely influenced by the gut microbiome. It is interesting to observe that both HBV-DC and HBV-ACLF patients have substantially more metabolites with higher Ratio_Port/Peri_ (250 and 123) than those with decreasing Ratio_Port/Peri_ (39 and 50) as compared to controls (Figure 5c), possibly due to microbiome dysbiosis and increased gut mucosa permeability in ACLF/DC progression. Higher Ratio_Port/Peri_ of microbial tryptophan metabolites (quinaldic acid and 2-aminomuconic acid) and other microbial metabolites (2,5-furandicarboxylic acid, pyridoxamine 5’-phosphate, indole-3-lactic acid, N-acetyl-tyrosine, and 3-sulfodeoxycholic acid) are commonly found in patients with HBV-ACLF and HBV-DC. We also observed decreased release of major BA conjugates (taurocholic acid and glycocholic acid) and enhanced release of 12-ketodeoxycholic acid in ACLF/DC patients. Interestingly, there were substantially more metabolites with lower Ratio_Port/Peri_ than with higher Ratio_Port/Peri_ (106 vs. 32) when comparing HBV-ACLF vs. HBV-DC patients, possibly due to the higher severity of portal hypertension in DC patients. These metabolites include the microbial metabolites, 3-methoxytyrosine, indolyl-3-acryloylglycine, pentosidine, and 3-methylene-indolenine.

### Serum oxlipidomic profile differentiate DC vs ACLF

**Figure 6.**
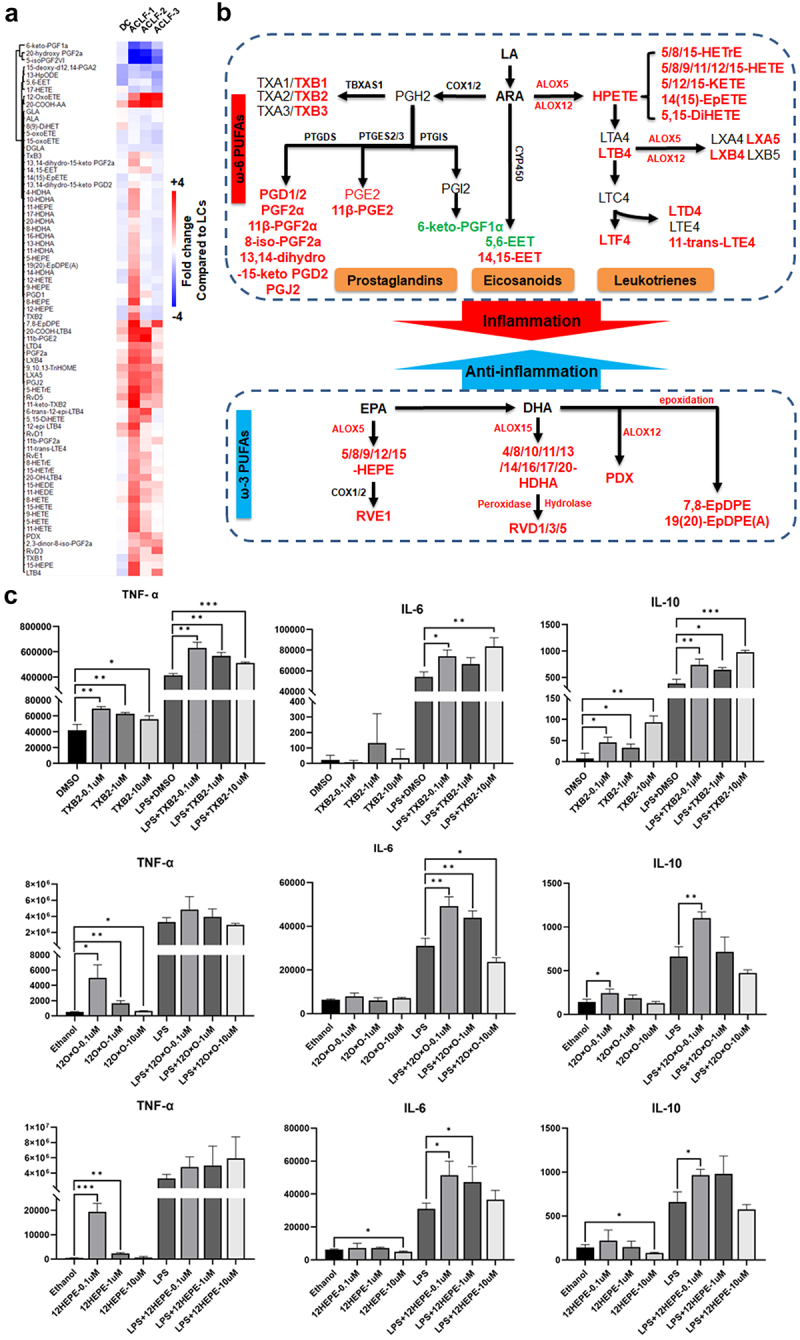
Alteration of circulating oxlipids between patients were summarized by heatmaps (a). Average log2 transformed level within each patient group were used. Up- or down-regulation as compared to LCs were colored by red or green shades. Proposed modulation of PUFA metabolism in ACLF patients (b). Significant upregulated or down-regulated species in ACLF grade 1 patients vs DC patients were colored in red or green.

To confirm the association of elevated oxlipids with extrahepatic manifestations of HBV-ACLF, we performed an oxlipidomic analysis of peripheral serum from 75 patients with HBV-ACLF (ACLF1, n = 54, ACLF2 n = 11, ACLF3 n = 10) and compared it with that of 20 DCs and 20 patients with liver cirrhosis (LC) (Supplementary Table S5). ACLF grade 1 patients shown a more profound upregulation in oxlipid profile, particularly of pro-inflammatory ω-6 PUFA derivatives (Figure 6a), including eicosanoids (8,9-EET), prostaglandins (PGD1/2, PGJ2, 11β-PGF2α, 11β-PGE2), leukotrienes (LTB4, LTD4, LTF4, 11-trans-LTE4), thromboxanes (TXB1/2/3), and their intermediate derivatives, including hydroxyl- eicosatetraenoic acids (5/8/9/11/12/15-HETE), dihydroxy- eicosatrienoic acid (5,15-DiHETE), and ketoeicosatetraenoic acid (5/12/15-KETE). In addition, higher levels of anti-inflammatory lipids, such as EPA-derived resolvin E1 and intermediate precursors (5/8/9/12/15-HEPE), DHA-derived protectin D1 (also known as 10,17-DiHDoHE), and resolvin D1/3/5, and their precursors 4/8/10/11/13/14/16/17/20-HDHA, were also recorded in ACLF grade 1 patients. These results suggest a possible feedback loop of anti-inflammatory signaling by ω-3 PUFAs during HBV-ACLF pathogenesis. Surprisingly, we found that the majority of oxlipids declined in ACLF grade 2-3 patients.

### Oxlipid intermediates amplify inflammatory response in monocyte model

The ARA-derived prostaglandins, leukotrienes, are well-documented as pro-inflammatory cascade signals in ACLF, but little is known about the roles of thromboxanes and ARA intermediates in the augmentation of the inflammatory reaction. To this end, we tested the dose-dependent immune response of THP-1 monocytes to TXB2, 12HEPE, and 12OxOETE. As shown in Figure 6c, each oxlipid alone was able to trigger upregulation of TNF-α, but not IL-6. However, upon pre-incubation with LPS, IL-6 levels can be further augmented by oxlipid supplements. For IL-10, only T×B2showed significant stimulation across the three concentrations, with or without LPS preincubation. When applied alone, either 12-KETE or 12-HEPE showed significant stimulation of TNF-α release only at low concentrations, and increased IL-6/IL-10 release at low concentrations with LPS.

## Discussion

The liver functions as the primary “biochemical factory” and contributes significantly to the blood metabolite pool. Therefore, fluctuations in the serum metabolome are expected in patients with a substantial loss of hepatic function. Indeed, the primary observation in this study was the drastic modulation of metabolites associated with bioenergetics, AAs, purines, and pyrimidine metabolism in ACLF/DC patients. The crippled hepatic metabolic functions were also evidenced by the highly correlated metabolomic profiles between the hepatic and portal veins in each ACLF/DC patient (Figure 2b).

In addition to hepatic function loss, extrahepatic factors also contribute substantially to serum metabolomic modulation in ACLF/DC patients. The pair-wise compartment ratio of each metabolite in each patient showed subtle changes related to extrahepatic events that are widely implicated in ACLF/DC pathogenesis. Compared to the limited differences in Ratio_Hep/Port_ of metabolites between HBV-DC and HBV-ACLF patients, HBV-ACLF patients had more metabolites with increasing Ratio_Peri/Hep_ and HBV-DC patients had more metabolites with increasing Ratio_Port/Peri_ (Figure 3c). These data implied that the HBV-DC and HBV-ACLF patients similarly suffered from hepatic function losses, but HBV-ACLF is complicated with more extrahepatic metabolic modulation, while DC patients have more gut microbiome-related modulation.

Among the complex metabolomic changes related to ACLF/DC pathogenesis, the AAs metabolism is the most overrepresented. Levels of proteinogenic AAs, including arginine, serine, glycine, methionine, proline, glutamine, phenylalanine, and tyrosine, were all higher in HBV-DC patients, and even higher in HBV-ACLF patients (Figure 4). These observations agree with those of recent reports in both Chinese HBV-ACLF cohort [21] and European ACLF cohort with cirrhosis [22]. Higher circulating AAs levels reflect impaired protein synthesis as well as profound catabolic processes and are also linked to propionic acidemia, hyperammonemia, sarcopenia, and hepatic encephalopathy commonly seen in ESLD patients, as well as cardiomyopathy and renal failure, which occasionally occur in ESLD patients [28–31]. In particular, glutamine is the primary vehicle of ammonia destined for hepatic detoxification via ureagenesis [32], and hyperglutaminemia has been linked to hepatic encephalopathy in ACLF of other etiologies [22,33]. Besides being crucial for ammonia detoxification, glutamine metabolism is also essential for bioenergetics [32]. This study also observed the downregulation of mitochondrial glutamate dehydrogenase (GLUD1/2, Figure 4b), which may lead to glutamine accumulation, respiratory chain dysfunction, and NADPH depletion.

Hypermethioninemia, a metabolic complication of impaired methionine elimination, is well known in patients with advanced cirrhosis and can lead to neurological and cardiovascular disorders [34]. We found that multiple enzymes in the methionine cycle were suppressed in HBV-ACLF patients, along with decreased availability of S-adenosyl-methionine (SAM), taurine, and glutathione (GSH), leading to reduced detoxification capability in the plasma. In contrast, enzymes in the methionine salvage pathway remained unchanged in ACLF patients, suggesting a higher metabolic flux toward the SAM-MTA-MTR route to generate more methionine and adenine required by drastic immune activities.

We also observed higher levels of arginine in the HBV-ACLF serum in all compartments (Figure 4c), in agreement with the ACLF of EASL study [22]. Hyperargininemia is likely due to accelerated protein degradation. Moreover, HBV-ACLF is characterized by a crippled urea cycle, as hepatocyte arginase 1 (ARG1), which converts arginine to urea, is downregulated in HBV-ACLF. Arginine is known to generate NO in response to inflammation and can directly affect the metabolic fitness and survival of T lymphocytes [35]. However, how hyperargininemia affects T-lymphocyte functionality in patients is still elusive. Moreover, patients with ACLF also have higher levels of homoarginine, which was previously found to be associated with cirrhosis [36].

This study also offered concrete evidence of oxlipid dysregulation in HBV-ACLF patients by both untargeted and targeted metabolomics in the two cohorts. HBV-ACLF patients had significantly higher levels of pro-inflammatory ω-6 PUFA than HBV-DC and control patients (Figure 6b). Interestingly, higher Ratio_Peri/Hep_ of eicosanoids in HBV-ACLF patients indicated that such changes were rooted in extrahepatic systemic inflammation, which is the main driving force of ACLF progression [15,37–39]. In addition to prostaglandins and eicosanoids, which are known to stimulate the production of inflammatory cytokines, we showed that the intermediate oxlipids generated can also amplify inflammation in monocytes (Figure 6c). It should be noted that the “eicosanoid storm” was only seen in early ACLF stage (Figure 6a), supporting the postulated transition from the early “‘pro-inflammatory’” phenotype to an immunodeficient phenotype. A recent study also found the enrichment of TREM2+ macrophages in the ACLF liver and its immunosuppressive phenotype was related to abnormal FFAs during ACLF onset [40]. This finding suggests a golden window that may benefit from novel therapies targeting systemic inflammation. Interestingly, a recent study also described overproduction of oxlipids in HBV-ACLF patients [21], and another recent study revealed that removing pro-inflammatory arachidonic acids by hemoperfusion adsorption is associated with favorable outcome in ACLF patients [41].

Previous studies have demonstrated that gut microbiota disorders were a prelude to the development of ACLF [14,42,43], and substantial changes in microbial-related metabolites correlated with disease severity and outcome in NACSELD-ACLF patients and HBV-ACLF have been reported [18,21]. Our study also found that HBV-ACLF/DC patients have higher Ratio_Port/Peri_ of microbial metabolites and toxins such as 3-methoxytyrosine and 3-sulfodeoxycholic acid [44,45]. We also observed decreased Ratio_Port/Peri_ of major BA conjugates (TCA and GCA) and enhanced Ratio_Port/Peri_ of 12-ketodeoxycholic acid due to impaired enterohepatic recycling of BA in ACLF/DC patients.

Several previous metabolomics studies have revealed variations in AAs and mitochondrial FA β-oxidation in ACLF of different etiologies [19–22], consistent with our observation in HBV-ACLF patients. Our study was limited by relatively small size of study cohort, and therefore we did not aim to develop metabolite biomarkers to differentiate ACLF from DC, as several previous studies have shown [19,20,23]. However, out of the 12 metabolites proposed previously as ACLF-signature that were also surveyed in this study [20], nine (Supplementary Figure S3) were significantly upregulated in HBV-ACLF patients (N-acetylaspartylglutamic acid, L-kynurenine, quinolinic acid, saccharopine, hexanoylcarnitine, phenol, N-acetyl-L-tyrosine, pantothenic acid, and N-acetyl-L-tryptophan). Importantly, hexanoylcarnitine, an indicator of mitochondrial dysfunction, was included in a recent metabolomic-based ACLF prognosis model [23] and N-acetylaspartylglutamic acid (NAAG) was considered as a prognosis indicator in a recent HBV-ACLF cohort study [21]. Given such similar traits, we postulate that these biomarkers can be used to monitor ACLF progression, regardless of underlying chronic etiologies. Importantly, although HBV-ACLF patients in this study were identified by COSSH criteria, we did select those patients with liver cirrhosis to meet the EASL criteria. Another important limitation of our results is the lack of metabolomic profile of different compartments from patients with CHB which is considered as the previous stage of HBV-DC/ACLF.

In summary, this study revealed hepatic metabolic malfunction in HBV-DC patients, which became more significant in HBV-ACLF patients and was mainly related to energy, FA, AA, and pyrimidine metabolism. Extrahepatic metabolic modulation stems from systemic inflammation, which is more evident in patients with HBV-ACLF, and metabolic modulation of gut microbiome dysbiosis, which is more evident in patients with HBV-DC. These findings support a synergy between liver-specific mechanisms and systemic inflammation in the development of ACLF/DCs. We further confirmed a pro-inflammatory oxlipid outburst in the early stages of HBV-ACLF development. This study provides a valuable resource for future mechanistic investigations of the metabolic modulations involved in ACLF/DC pathogenesis and biomarker development for ACLF/DC management.

## Supplementary Material

Suppl_Table4.xlsx

Suppl_Table3.xlsx

Fig_S2.tif

Suppl_Table5.xlsx

Fig_S3.tif

Fig_S1.tif

Suppl_Table1.xlsx

Suppl_Table2.xlsx

## Data Availability

The data that support the findings of this study are openly available in: https://pan.zju.edu.cn/share/d0e76066639c5cb5dfebefd980
