# Supplementary figures and images for: Hepatic and extrahepatic metabolic modulation in hbv-related decompensated cirrhosis and acute-on-chronic liver failure
Source: Virulence. 2024 Sep 23;15(1):2404953. doi: 10.1080/21505594.2024.2404953 (PMC11421379; doi:10.1080/21505594.2024.2404953)

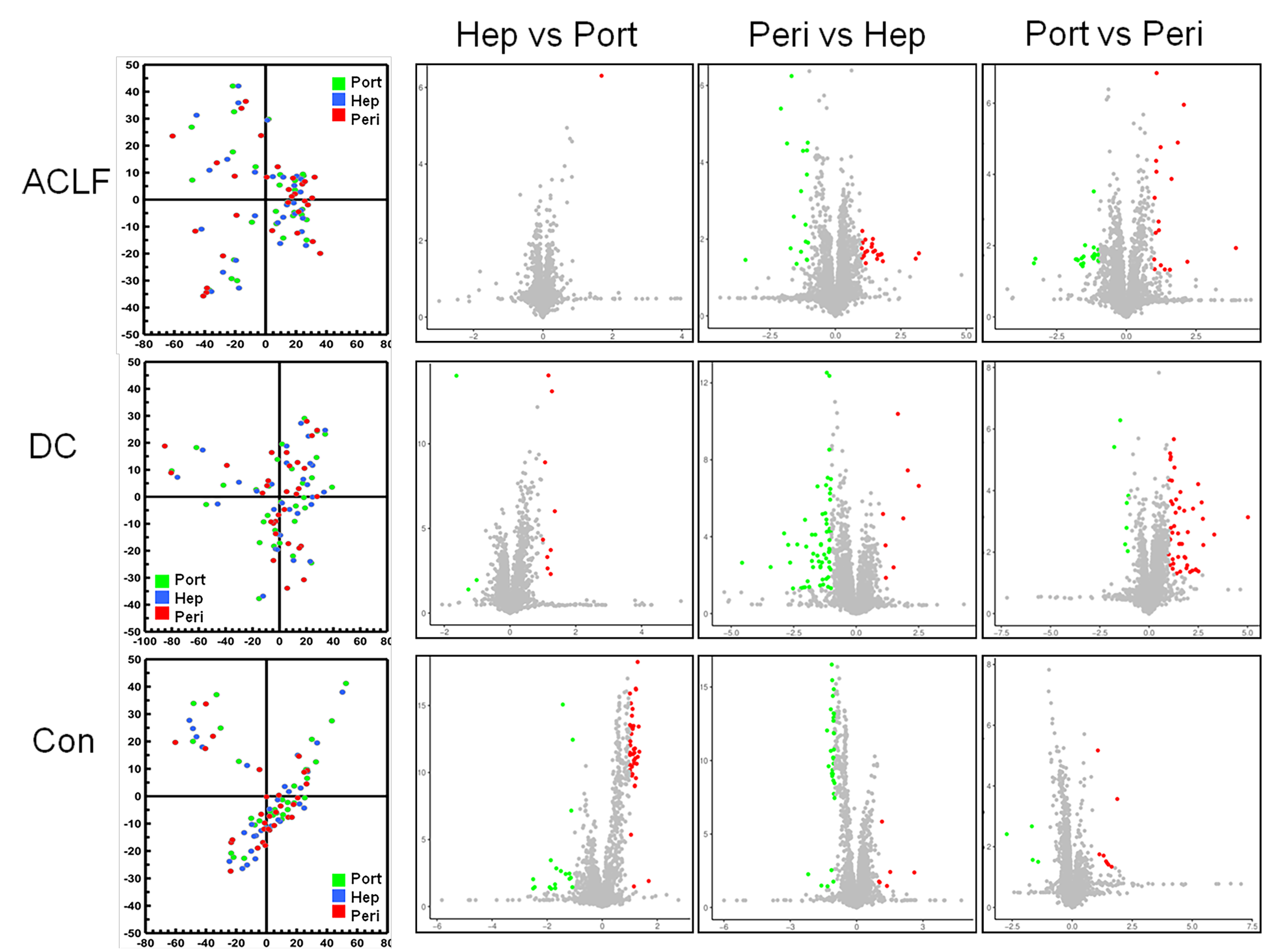

Supplement: Fig_S2.tif [file KVIR_A_2404953_SM9983.tif]

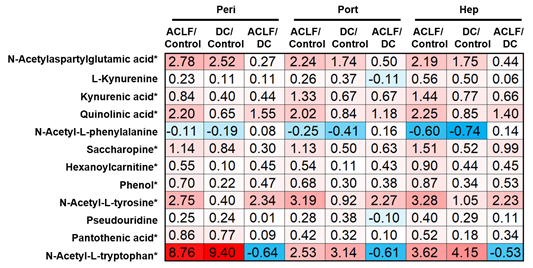

Supplement: Fig_S3.tif [file KVIR_A_2404953_SM9981.tif]

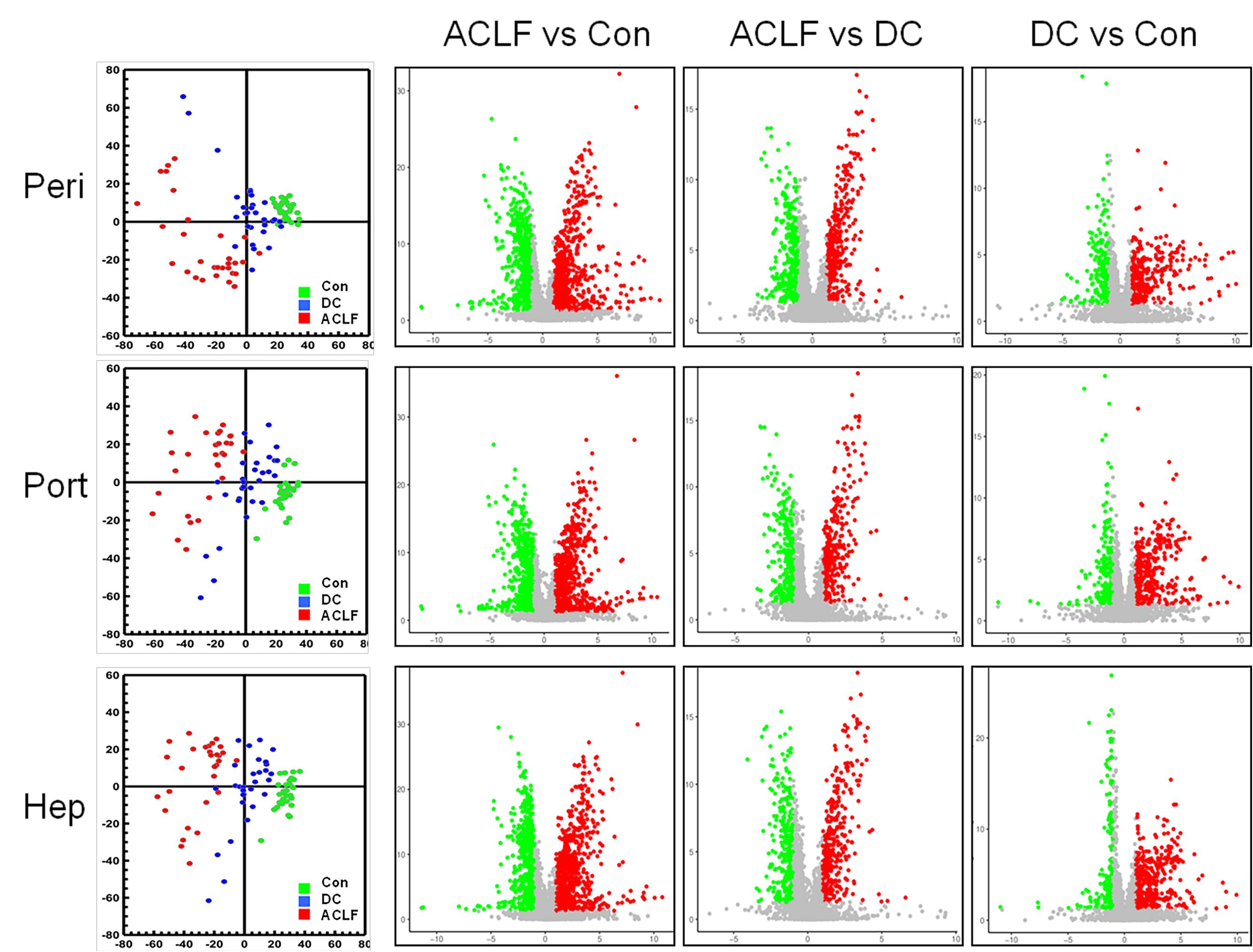

Supplement: Fig_S1.tif [file KVIR_A_2404953_SM9980.tif]
